# Changes in lumbar lordosis and predicted minimum 5-year surgical outcomes after short-segment transforaminal lumbar interbody fusion

**DOI:** 10.1038/s41598-022-18679-7

**Published:** 2022-08-23

**Authors:** Yasuchika Aoki, Masahiro Inoue, Hiroshi Takahashi, Arata Nakajima, Masato Sonobe, Fumiaki Terajima, Takayuki Nakajima, Yusuke Sato, Go Kubota, Masashi Sato, Satoshi Yoh, Shuhei Ohyama, Junya Saito, Masaki Norimoto, Yawara Eguchi, Sumihisa Orita, Kazuhide Inage, Yasuhiro Shiga, Seiji Ohtori, Koichi Nakagawa

**Affiliations:** 1 Department of Orthopaedic Surgery, Eastern Chiba Medical Center, 3-6-2 Okayamadai, Togane, Chiba 283-8686 Japan; 2 Department of General Medical Science, Graduate School of Medicine, Chiba University, Chiba, Chiba Japan; 3 Department of Orthopaedic Surgery, University of Tsukuba, Tsukuba, Ibaraki Japan; 4 Department of Orthopaedic Surgery, Toho University Sakura Medical Center, Sakura, Chiba Japan; 5 Department of Orthopaedic Surgery, Kubota Orthopaedic Clinic, Katori, Chiba Japan; 6 Department of Orthopaedic Surgery, Graduate School of Medicine, Chiba University, Chiba, Chiba Japan

**Keywords:** Medical research, Outcomes research

## Abstract

Although most patients who undergo transforaminal lumbar interbody fusion (TLIF) show favorable surgical results, some still have unfavorable results for various reasons. This study aimed to investigate the influence of differences in lumbar lordosis (LL) between the standing and supine positions (DiLL: supine LL–standing LL) on minimum 5-year surgical outcomes after short-segment TLIF. Ninety-one patients with lumbar degenerative disease who underwent short-segment TLIF (1–2 levels) were categorized based on preoperative differences in LL as DiLL (+) and DiLL (−). Comparison and correlation analyses were performed. The incidence of adjacent segment disease (ASD) by radiology (R-ASD) and symptomatic ASD (S-ASD), bony fusion rates, and pre- and postoperative clinical scores (visual analog scale [VAS]; Japanese Orthopaedic Association [JOA] score; Oswestry disability index (ODI); and Nakai’s score) were evaluated. Postoperatively, VAS for low back pain (LBP) in the sitting position, JOA scores for LBP, lower leg pain, intermittent claudication, ODI, and Nakai’s score were significantly worse in the DiLL (+) group than in the DiLL (−) group. DiLL values were significantly correlated with VAS for LBP, ODI, and Nakai’s score, postoperatively. Positive DiLL values were associated with poorer postoperative outcomes. DiLL is a simple and useful method for predicting mid-term outcomes after TLIF.

## Introduction

Lumbar spinal fusion surgery is a common treatment for various pathologies of the lumbar spine. Transforaminal lumbar interbody fusion (TLIF) is a standard lumbar spinal fusion procedure used to treat degenerative lumbar diseases^1–4^. Although most patients who undergo TLIF show favorable surgical results, some patients still have unfavorable results for various reasons such as residual low back and lower-extremity symptoms, adjacent segment disease (ASD), pseudoarthrosis, sagittal malalignment, and surgical complications^5–8^. Accordingly, several studies have been conducted to identify the predictive factors of clinical outcomes after TLIF^8–12^. Predictors of clinical outcomes after TLIFremain controversial; thus, further research is required to establish a consensus.

The preoperative postural difference in lumbar lordosis (LL) between the standing and supine positions (DiLL) has recently been reported to be correlated with short-term postoperative clinical outcomes after single-level TLIF^13^. The report concluded that patients with higher preoperative DiLL values tended to show worse postoperative residual symptoms such as lower extremity symptoms, low back pain (LBP) upon standing, and gait disturbance 2 years after TLIF^13^. However, mid- to long-term postoperative clinical outcomes relative to the preoperative DiLL have not been reported. Therefore, we conducted a retrospective study to evaluate the minimum 5-year clinical outcomes for patients with lumbar degenerative disease who underwent short-segment TLIF. This study aimed to examine how preoperative DiLL influences the incidences of ASD, bony fusion rate, and preoperative and 5-year postoperative clinical outcomes.

## Materials and methods

**Figure 1:**
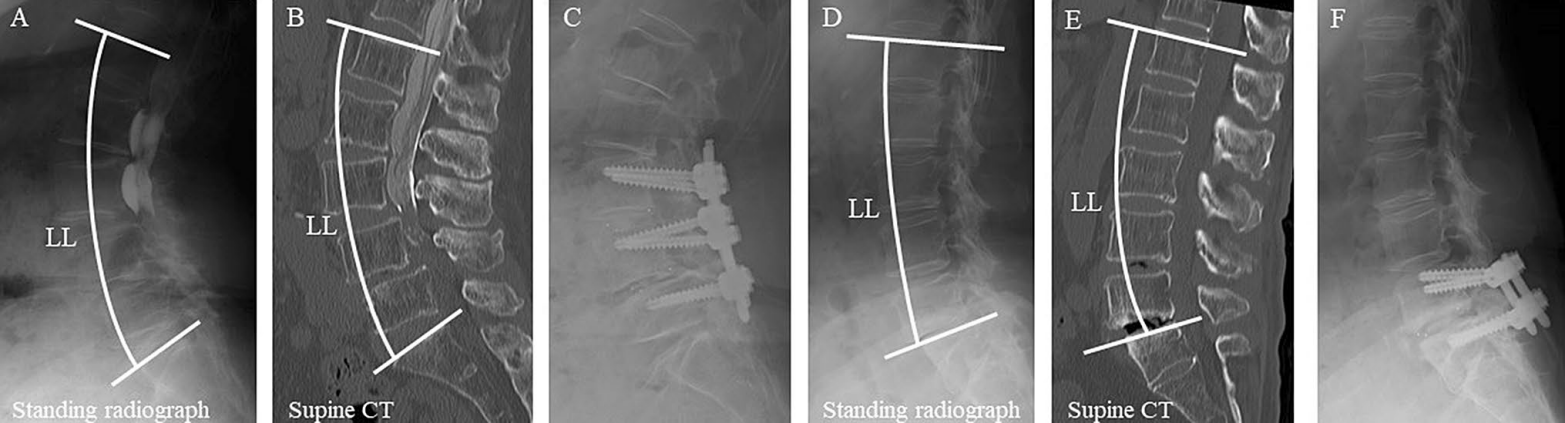
Pre-operative lateral radiographs obtained in the standing position (**A**,**D**), computed tomography (CT) images obtained in the supine position (**B**,**E**), and postoperative lateral radiographs taken after short-segment transforaminal lumbar interbody fusion (**C**,**F**) of DiLL (−) patient (**A**–**C**, DiLL < 0°) and DiLL (+) patient (**D**–**F**, DiLL ≥ 0°). In DiLL (−) patient, the lumbar lordosis (LL) is greater in the standing radiograph (**A**) than in the supine CT (**B**), while the LL is smaller in the standing radiograph (**D**) than in the supine CT (**E**). *DiLL* difference in lumbar lordosis between the standing and supine positions.

The study was conducted in accordance with the Declaration of Helsinki, and the study protocol was approved by the institutional review board of our medical center. Preoperative demographic data, including age, sex, and body mass index (BMI) were collected. All patients provided informed consent prior to the surgery. The clinical records of consecutive patients with lumbar degenerative disease including lumbar degenerative spondylolisthesis, lumbar foraminal stenosis, spondylolytic spondylolisthesis, lateral lumbar disc herniation, lumbar spinal stenosis, and lumbar facet joint cysts, who underwent short-segment TLIF^9^ (Fig. 1) at one or two levels at our hospital between August 2010 and November 2016 were retrospectively reviewed. All surgeries were performed by four spine surgeons, each with over 10 years of clinical experience. Patients with a history of other fusion procedures (such as posterolateral fusion or oblique lateral lumbar interbody fusion combined with TLIF) and patients with any indication of other pathological conditions, such as infectious diseases, malignant neoplasms, or significant trauma were excluded and 137 patients were included. During the 5-year follow-up, 6 patients died and 4 patients had diseases affecting activities of daily living, such as Parkinson’s disease (2 cases), cerebral infarction (1 case), or cerebral hemorrhage (1 case). Thirty-six patients were unable to complete the 5-year follow-up. The final analysis included a total of 91 patients.

### Evaluation of clinical outcomes

Postoperative data were reviewed to determine whether the patients had postoperative ASD or not. Radiological ASD (R-ASD) of both cranial and caudal adjacent discs was evaluated at the 5-year follow-up. When there was slippage progression of > 3 mm, a posterior opening of > 5°, and a narrowing of the disc height of > 3 mm in comparison with preoperative flexion–extension lateral radiographs, patients were considered to have R-ASD, as described in previous studies^14,15^. Postoperative symptomatic ASD (S-ASD) was diagnosed when spinal canal stenosis, foraminal stenosis, disc herniation, or segmental kyphosis (> 5°) was present at segments adjacent to the operated segments and low back or lower leg symptoms were obviously caused by ASD. Patients were considered to have S-ASD when the symptoms persisted for at least three months and required additional treatment, including medications, epidural blockades, or subsequent surgeries. Bony fusion was evaluated using radiographs and computed tomography (CT) images 1 year postoperatively, and the evaluation was performed repeatedly until bony fusion was confirmed or until the final follow-up. Fusion was defined as (i) the presence of continuous trabecular bone formation through or outside the cages, (ii) < 3° movement on lateral flexion and extension radiographs, and (iii) the absence of radiolucent lines of > 50% of the implant^16–19^. In patients with two-level fusion, we considered that fusion was achieved when both levels achieved bony fusion. Clinical outcomes were assessed using the (1) visual analog scale (VAS) for LBP, lower-extremity pain and numbness between 0 (no pain) and 10 (maximal pain); (2) our originally developed detailed VAS scoring system for LBP in motion, standing position, and sitting position^20^; (3) Japanese Orthopaedic Association (JOA) scores for LBP, lower-extremity pain, and intermittent claudication (Table 1); (4) Oswestry disability index (ODI); and (5) Nakai’s scoring system for the evaluation of surgical outcomes, in which scores were classified as excellent (3), good (2), fair (1), or poor (0) (Table 1)^21^. Clinical scores were evaluated preoperatively and at 5 years postoperatively. Postoperative improvement in clinical scores was calculated by comparing the pre- and postoperative clinical scores. Three patients reqiuired a revision surgery within 5 years of the TLIF and 14 patients who did not complete the questionnaires either preoperatively or at 5 years postoperatively were excluded. Finally, 74 patients were included for the evaluation of clinical scores.

**Table 1:** Clinical scoring systems.

**JOA score for low back pain**	
None	3
Occasional	2
Frequent mild or occasional severe pain	1
Frequent or continuous severe pain	0
**JOA score for lower leg pain and tingling**	
None	3
Occasional slight or severe symptom	2
Frequent slight or occasional severe symptom	1
Frequent or continuous severe symptom	0
**JOA score for intermittent claudication**	
Normal	3
Able to walk > 00 m, although it causes tingling and/or muscle weakness	2
Unable to walk > 500 m due to leg pain, tingling, and/or muscle weakness	1
Unable to walk > 100 m due to leg pain, tingling, and/or muscle weakness	0
**Nakai’s score**	
Patient has resumed work-related and other activities with slight or no symptoms	3: Excellent
Patient has resumed work-related and other activities but occasionally feels pain in the back or lower limbs after strenuous work	2: Good
Patient has reduced work-related and other activities due to residual pain in the back or lower limbs	1: Fair
Patient cannot work or carry out activities of daily living and is considered disabled	0: Poor

### Preoperative radiological evaluation

Lumbopelvic parameters, such as LL (the angle between the superior endplates of L1 and S1) and pelvic incidence (PI, the angle between a line perpendicular to the sacral plate at its midpoint and the line connecting the hip axis that connected the centers of both femoral heads and the sacral end plate midpoint) were measured using preoperative lateral radiographs obtained with the patient in the standing position (Fig. 1A). In addition, LL in the supine position was measured using preoperative sagittal reconstruction CT images (Fig. 1B). The DiLL between the standing and supine positions was calculated as the supine LL–standing LL. Preoperative anteroposterior standing radiographs were used to examine the coronal Cobb angle at the levels between the T10 and S1.

### Demographic data and clinical outcomes

Patients with DiLL ≥ 0° were defined as DiLL (+), and those with DiLL < 0° were defined as DiLL (−) (Fig. 1). The patients’ preoperative data, including age, sex, body mass index (BMI), preoperative lumbopelvic parameters (including supine LL, standing LL, and PI-LL [pelvic incidence minus lumbar lordosis]), lumbar flexibility (difference in LL between flexion and extension positions), scoliosis, number and levels of fused segments were compared between the two groups. The incidence of postoperative R-ASD and S-ASD, bony fusion rate, pre- and postoperative VAS, JOA, and ODI scores, postoperative improvement in VAS, JOA, and ODI scores; and postoperative Nakai’s score were compared between the two groups. Furthermore, the above-mentioned clinical data were compared between the DiLL (+) and DiLL (−) groups after excluding patients with scoliosis (> 10°).

### Examining the correlation between preoperative DiLL and clinical outcomes

Correlation analysis was performed between the preoperative DiLL and R-ASD, S-ASD, and bony fusion status at 1 and 5 years, postoperatively, and each clinical score to examine the association between preoperative DiLL and postoperative clinical outcomes. To exclude the influence of age, sex, BMI, presence of scoliosis, and the number of fused segments, multiple regression analysis was performed after appropriate adjustments.

### Examining the influence of DiLL on clinical outcomes in patients with or without preoperative PI-LL mismatch

To examine the influence of DiLL on surgical outcomes in patients with or without PI-LL mismatch, patients were divided into two groups based on preoperative standing radiographs: mismatched (PI-LL > 10°) and matched (PI-LL ≤ 10°) subgroups. Clinical outcomes were compared between DiLL (+) and DiLL (−) patients in each group (mismatched and matched).

### Data analyses

Continuous data are presented as the mean ± standard deviation, and categorical variables are presented as numbers. The age, BMI, lumbopelvic parameters, VAS, and ODI of the two groups were compared using an unpaired t-test. Pearson’s chi-square test (or Yates’ chi-square test when any expected frequencies were < 5) was used to compare sex differences and percentages of patients with scoliosis, single-level fusion, R-ASD, S-ASD, and bony fusion rates. The Mann–Whitney U test was used to compare JOA and Nakai’s scores. To investigate the relative influence of preoperative DiLL on pre and postoperative clinical outcomes, such as VAS, JOA scores, ODI, and Nakai’s score, a multiple regression analysis was performed after adjustment for age, sex, BMI, presence of scoliosis, and number of fused segments, with DiLL as the independent variable and each clinical outcome as the dependent variable. Statistical significance was set at p < 0.05.

### Ethical declarations

The study was conducted in accordance with the Declaration of Helsinki, and the study protocol was approved by the institutional review board of Toho University Sakura Medical Center. (No. 2012–071).

### Consent to participate/consent to publish

All patients provided informed consent prior to surgery.

## Results

The DiLL (+) group included 48 patients and the DiLL (−) group included 43 patients (Table 2). The DiLL (+) group (69.7 ± 7.7 years old) was significantly older than the DiLL (−) group (64.0 ± 13.0 years old) (p = 0.015); however, no significant differences in sex or BMI were found between the two groups. The mean preoperative PI was not significantly different. However, the preoperative supine LL (p = 0.0094) and standing LL (p < 0.001) were significantly smaller in the DiLL (+) group than in the DiLL (−) group. Thus, the mean preoperative PI-LL (p < 0.001) was significantly greater in the DiLL (+) group than in the DiLL (−) group. Lumbar flexibility was not significantly different between the two groups. The percentage of patients with scoliosis was significantly greater in the DiLL (+) group than in the DiLL (−) group. Three patients in the DiLL (+) group showed scoliotic curvature > 20°, but none in the DiLL (−) group. The mean coronal Cobb angle was significantly greater in the DiLL (+) group than in the DiLL (−) group, and there was no significant difference in the number of fused segments or in the number of patients who received L5-S1 fusion between the two groups. The mean follow-up period was 81.5 ± 18.6 months (range 60–130 months).

**Table 2:** Demographic data and radiological outcomes.

	DiLL (+)	DiLL (−)	p
Number of patients	48	43	–
Age (years)	69.7 ± 7.7	64.0 ± 13.0	0.015*
Sex (male/female)	24/24	15/28	0.15
Body mass index (kg/m^2^)	25.2 ± 4.0	24.4 ± 3.4	0.27
Supine LL (°)	32.4 ± 13.1	39.0 ± 10.3	0.0094*
Standing LL (°)	24.5 ± 14.4	44.8 ± 9.8	< 0.001*
PI (°)	47.3 ± 8.7	50.7 ± 9.1	0.076
PI-LL (°)	22.8 ± 15.4	5.9 ± 10.6	< 0.001*
Lumbar flexibility (°)	28.4 ± 12.2	30.3 ± 13.2	0.48
Scoliosis (> 10°)	22	10	0.024*
Scoliosis (> 20°)	3	0	0.28
Coronal Cobb angle (°)	10.0 ± 7.1	6.7 ± 4.7	0.0098*
Number of fused segments (1 level/2 levels)	33/15	34/9	0.26
Level of fused segment	L2–L3: 3 L3–L4: 14 L4–L5: 35 L5–S1: 9 L5–L6: 2	L2–L3: 0 L3–L4: 9 L4–L5: 37 L5–S1: 4 L5–L6: 1	
R-ASD (%)	25.0%	16.3%	0.31
S-ASD (%)	22.9%	7.0%	0.070
Bony fusion rate (1 year)	60.4%	72.1%	0.24
Bony fusion rate (5 years)	97.8%	97.6%	0.52

Continuous data are presented as mean ± standard deviation. Categorical data are presented as numbers.

Asterisks indicate statistically significant differences (p < 0.05).

*LL* lumbar lordosis, *DiLL* difference in preoperative LL (supine LL–standing LL), *PI* pelvic incidence, *Lumbar flexibility* difference in LL between flexion and extension positions; *R-ASD* radiological adjacent segment disease, *S-ASD* symptomatic adjacent segment disease.

### Influence of DiLL on clinical outcomes

There was no significant difference in the percentage of patients with postoperative R-ASD between the DiLL (+) and DiLL (−) groups. In the DiLL (+) group, 11 patients (22.9%) had postoperative S-ASD (Table 2), including five who underwent revision surgery [lumbar spinal stenosis (n = 3), disc herniation (n = 1), and severe LBP due to adjacent segment kyphosis (n = 1)]. Two of the five patients underwent revision surgery within 5 years after TLIF. In the DiLL (−) group, three patients had postoperative ASD, including two who underwent revision surgery for lumbar spinal stenosis (n = 1) and foraminal stenosis (n = 1). One of the two patients underwent revision surgery within 5 years after TLIF. Accordingly, these three patients were excluded from the analysis of clinical outcomes evaluated 5 years, postoperatively. The number of patients with postoperative S-ASD was higher in the DiLL (+) group (22.9%: 11/48) than in the DiLL (−) group (7.0%: 3/43); however, the difference was not significant. No significant difference in bony fusion rates at 1 and 5 years postoperatively was observed between the DiLL (+) and DiLL (−) groups (Table 2).

The preoperative clinical scores were not significantly different between the two groups (Table 3). The postoperative VASs scores for LBP, lower leg pain, and lower leg numbness were not significantly different between the two groups; however, our originally developed detailed VAS for LBP revealed that LBP in the sitting position was significantly greater in the DiLL (+) group than in the DiLL (−) group. The postoperative JOA scores for LBP, lower-extremity pain, intermittent claudication, ODI, and Nakai’s scores were significantly worse in the DiLL (+) group than in the DiLL (−) group (Table 3). The analysis of postoperative improvement in each score revealed that the DiLL (−) group demonstrated better postoperative improvement in the JOA score for LBP and lower-extremity pain than the DiLL (+) group (Table 3).

**Table 3:** Pre and postoperative visual analog scale (VAS), Japanese Orthopaedic Association (JOA) score, Oswestry disability idex (ODI), and Nakai’s score.

		DiLL (+)	DiLL (−)	p
**Preoperative**				
VAS	Low back pain (LBP)	6.1 ± 2.7	5.6 ± 2.3	0.35
	Lower leg pain	6.9 ± 2.7	7.2 ± 2.1	0.64
	Lower leg numbness	6.9 ± 2.6	6.1 ± 3.1	0.23
Detailed VAS	LBP in motion	5.5 ± 3.4	5.5 ± 3.0	0.98
	LBP in standing	7.1 ± 3.2	6.8 ± 2.7	0.72
	LBP in sitting	4.5 ± 3.1	4.7 ± 2.5	0.79
JOA Score	LBP	1.2 ± 0.7	1.1 ± 0.6	0.47
	Lower leg pain	0.7 ± 0.6	0.5 ± 0.6	0.14
	Intermittent claudication	0.6 ± 0.8	0.8 ± 0.8	0.19
ODI		47.1 ± 19.0	41.9 ± 15.2	0.20
**Postoperative**				
VAS	LBP	2.5 ± 2.4	1.7 ± 2.3	0.17
	Lower leg pain	2.3 ± 2.7	2.1 ± 2.5	0.71
	Lower leg numbness	2.4 ± 2.8	1.9 ± 2.4	0.43
Detailed VAS	LBP in motion	2.1 ± 2.3	1.6 ± 2.1	0.37
	LBP in standing	2.8 ± 2.5	1.8 ± 2.0	0.086
	LBP in sitting	2.3 ± 2.7	1.0 ± 1.5	0.013*
JOA Score	LBP	2.2 ± 0.8	2.6 ± 0.5	0.010*
	Lower leg pain	2.2 ± 0.7	2.6 ± 0.7	0.014*
	Intermittent claudication	2.3 ± 0.8	2.8 ± 0.5	0.0042*
ODI		25.6 ± 21.6	17.0 ± 14.1	0.050*
Nakai’s score		2.1 ± 0.9	2.7 ± 0.4	0.0013*
**Improvement (∆)**				
VAS	LBP	3.6 ± 3.6	3.8 ± 3.0	0.78
	Lower leg pain	4.6 ± 3.2	5.1 ± 3.5	0.53
	Lower leg numbness	4.5 ± 3.7	4.2 ± 3.9	0.72
Detailed VAS	LBP in motion	3.4 ± 3.6	3.9 ± 3.4	0.55
	LBP in standing	4.3 ± 4.2	5.0 ± 3.1	0.43
	LBP in sitting	2.2 ± 4.4	3.6 ± 2.7	0.088
JOA Score	LBP	0.9 ± 0.9	1.6 ± 0.8	0.0039*
	Lower leg pain	1.5 ± 1.0	2.1 ± 1.0	0.0070*
	Intermittent claudication	1.8 ± 1.1	2.0 ± 0.9	0.37
ODI		21.6 ± 26.6	24.9 ± 19.5	0.54

Data are presented as mean ± standard deviation.

Asterisks indicate statistically significant differences (p < 0.05).

Postoperative clinical scores were calculated at 5 years postoperatively.

After excluding patients with scoliosis (> 10°), similar results to the above-mentioned analysis were obtained regarding the difference in clinical outcomes between the DiLL (+) and DiLL (−) group (Table 4). Patients with scoliosis (> 10°) showed a non-significant tendency (p = 0.094) toward a higher incidence of S-ASD (8/31 cases, 25.8%) than those without scoliosis (6/60 cases, 10.0%). Among patients without scoliosis, the DiLL (+) group showed a higher incidence of S-ASD (5/26 cases, 19.2%) than the DiLL (−) group (1/34 cases, 2.9%), although the difference was not significant (p = 0.091). The DiLL (+) group exhibited worse postoperative VAS for LBP in sitting, JOA scores (LBP and intermittent claudication), and Nakai’s score, and less improvement in JOA scores (LBP and lower leg pain) than the DiLL (−) group.

**Table 4:** Pre and postoperative visual analog scale (VAS), Japanese Orthopaedic Association (JOA) score, Oswestry disability idex (ODI), and Nakai’s score in patients without scoliosis (> 10°).

		DiLL (+)	DiLL (−)	p
R-ASD (%)		23.1%	14.3%	0.58
S-ASD (%)		19.2%	2.9%	0.091
**Preoperative**				
VAS	Low back pain (LBP)	6.2 ± 2.8	5.2 ± 2.2	0.19
	Lower leg pain	6.9 ± 2.7	7.1 ± 2.0	0.75
	Lower leg numbness	7.2 ± 2.5	6.3 ± 2.8	0.26
Detailed VAS	LBP in motion	4.9 ± 3.3	5.4 ± 3.0	0.61
	LBP in standing	6.9 ± 3.2	6.7 ± 2.7	0.86
	LBP in sitting	4.5 ± 3.2	4.5 ± 2.4	0.98
JOA Score	LBP	1.3 ± 0.7	1.1 ± 0.7	0.35
	Lower leg pain	0.8 ± 0.6	0.5 ± 0.6	0.21
	Intermittent claudication	0.6 ± 0.7	0.8 ± 0.8	0.33
ODI		46.8 ± 22.1	41.9 ± 16.0	0.40
**Postoperative**				
VAS	LBP	2.7 ± 2.3	1.8 ± 2.3	0.21
	Lower leg pain	2.3 ± 2.7	2.3 ± 2.6	0.97
	Lower leg numbness	2.8 ± 3.2	2.1 ± 2.5	0.40
Detailed VAS	LBP in motion	2.0 ± 2.2	1.6 ± 2.1	0.54
	LBP in standing	3.2 ± 2.5	2.0 ± 2.0	0.090
	LBP in sitting	2.6 ± 3.0	1.1 ± 1.4	0.040*
JOA Score	LBP	2.1 ± 0.7	2.7 ± 0.5	0.0053*
	Lower leg pain	2.2 ± 0.7	2.5 ± 0.8	0.066
	Intermittent claudication	2.4 ± 0.7	2.8 ± 0.5	0.029*
ODI		25.3 ± 22.6	17.6 ± 13.6	0.19
Nakai’s score		2.1 ± 0.8	2.8 ± 0.4	0.0011*
**Improvement (∆)**				
VAS	LBP	3.5 ± 3.7	3.4 ± 2.7	0.90
	Lower leg pain	4.6 ± 3.4	4.8 ± 3.4	0.84
	Lower leg numbness	4.4 ± 4.0	4.2 ± 3.9	0.89
Detailed VAS	LBP in motion	2.9 ± 3.4	3.8 ± 3.3	0.39
	LBP in standing	3.7 ± 4.4	4.7 ± 3.0	0.38
	LBP in sitting	1.9 ± 4.6	3.4 ± 2.7	0.19
JOA Score	LBP	0.8 ± 0.8	1.6 ± 0.9	0.0030*
	Lower leg pain	1.4 ± 1.0	2.0 ± 1.0	0.048*
	Intermittent claudication	1.9 ± 1.0	2.0 ± 0.9	0.64
ODI		21.6 ± 29.9	24.4 ± 21.2	0.72

Data are presented as mean ± standard deviation.

Asterisks indicate statistically significant differences (p < 0.05).

Postoperative clinical scores were calculated at 5 years postoperatively.

### Correlation between DiLL and postoperative clinical outcomes

The correlation between the preoperative DiLL and postoperative outcomes was evaluated using multiple regression analysis (Table 5). After adjusting for age, sex, BMI, presence of scoliosis (> 10°), and number of fused segments, preoperative DiLL was not significantly correlated with R-ASD, S-ASD, or bony fusion rates at 1 and 5 years postoperatively. Preoperative DiLL was significantly corelated with postoperative VAS for LBP, and a detailed VAS scoring system revealed that preoperative DiLL was significantly correlated with LBP in the standing and sitting positions but was not correlated with LBP in motion. Two of the three JOA scores (LBP and intermittent claudication), ODI, and Nakai’s score were significantly correlted with preoperative DiLL. Generally, our results indicate that postoperative clinical outcomes were worse when the preoperative DiLL value was higher.

**Table 5:** Correlation between preoperative DiLL and postoperative clinical outcomes adjusted for age, sex, body mass index, scoliosis, and number of fused segments.

Dependent variables	Independent variable	Regression coefficient	Standardized regression coefficient	t-value	p-value
R-ASD	DiLL	0.004	0.093	0.804	0.42
S-ASD	DiLL	0.006	0.154	1.321	0.19
Bony fusion (1 year)	DiLL	− 0.003	− 0.063	0.560	0.58
Bony fusion (5 years)	DiLL	0.001	0.058	0.480	0.63
**VAS**					
Low back pain (LBP)	DiLL	0.071	0.273	2.074	0.042*
Lower leg pain	DiLL	0.018	0.064	0.474	0.64
Lower leg numbness	DiLL	0.023	0.079	0.598	0.55
LBP in motion	DiLL	0.057	0.232	1.722	0.089
LBP in standing	DiLL	0.079	0.303	2.393	0.019*
LBP in sitting	DiLL	0.092	0.365	2.845	0.0058*
**JOA scores**					
LBP	DiLL	− 0.038	− 0.489	4.101	< 0.001*
Lower leg pain	DiLL	− 0.021	− 0.250	1.897	0.062
Intermittent claudication	DiLL	− 0.035	− 0.462	3.889	< 0.001*
ODI	DiLL	0.650	0.312	2.450	0.017*
Nakai’s score	DiLL	− 0.041	− 0.495	4.123	< 0.001*

Asterisks indicate statistically significant differences (p < 0.05).

*R-ASD* radiological adjacent segment disease, *S-ASD* symptomatic adjacent segment disease, *LL* lumbar lordosis, *DiLL* difference in preoperative LL (supine LL–standing LL), *JOA score* Japanese Orthopaedic Association score, *ODI* Oswestry disability index.

### Influence of DiLL on clinical outcomes in patients with or without preoperative PI-LL mismatch

In the PI-LL mismatched patients, postoperative S-ASD was more likely to occur in the DiLL (+) group (9/38 patients) than in the DiLL (−) group (0/14 patients); however, the difference was not significant (p = 0.11, Table 6). None of the preoperative clinical scores (VASs, JOA scores, and ODI) showed significant difference between the DiLL (+) and DiLL (−) groups. Patients in the DiLL (+) group had significantly worse postoperative clinical outcomes on an item of the JOA score (intermittent claudication p = 0.0038). ODI (p = 0.050) and Nakai’s score (p = 0.0089) compared with patients in the DiLL (−) group. Postoperative improvement in the JOA score for LBP was significantly worse in the DiLL (+) group than in the DiLL (−) group (Table 6).

**Table 6:** Clinical outcomes in patients with or without PI-LL mismatch.

	Mismatched (PI-LL > 10°)			Matched (PI-LL ≤ 10°)		
	DiLL (+)	DiLL (−)	p	DiLL (+)	DiLL (−)	p
Number of patients	38	14	–	10	29	–
R-ASD (%)	28.9%	35.7%	0.90	10.0%	6.9%	0.71
S-ASD (%)	23.7%	0.0%	0.11	20.0%	10.3%	0.81
Bony fusion rate (1 year)	52.6%	71.4%	0.37	90.0%	72.4%	0.48
Bony fusion rate (5 years)	97.2%	100%	0.62	100%	96.4%	0.59
**Preoperative**						
**VAS**						
Low back pain (LBP)	6.0 ± 2.8	5.6 ± 2.6	0.70	6.9 ± 2.2	5.6 ± 2.2	0.23
Lower leg pain	6.8 ± 2.8	7.1 ± 2.6	0.75	7.1 ± 2.1	7.2 ± 1.9	0.95
Lower leg numbness	7.0 ± 2.7	5.6 ± 2.9	0.19	6.7 ± 2.3	6.3 ± 3.2	0.75
LBP in motion	5.4 ± 3.3	5.5 ± 3.2	0.95	5.6 ± 3.4	5.5 ± 2.9	0.95
LBP in standing	6.9 ± 3.3	6.6 ± 3.0	0.77	7.9 ± 2.7	7.0 ± 2.6	0.48
LBP in sitting	4.3 ± 3.2	4.5 ± 2.3	0.86	5.1 ± 2.3	4.7 ± 2.6	0.76
**JOA score**						
LBP	1.2 ± 0.7	1.1 ± 0.7	0.75	1.3 ± 0.5	1.1 ± 0.6	0.47
Lower leg pain	0.7 ± 0.6	0.5 ± 0.5	0.28	0.7 ± 0.5	0.5 ± 0.6	0.36
Intermittent claudication	0.5 ± 0.7	0.5 ± 0.8	0.99	0.7 ± 0.9	0.9 ± 0.8	0.59
ODI	49.0 ± 19.1	44.1 ± 18.3	0.48	38.9 ± 16.6	40.9 ± 13.5	0.78
**Postoperative**						
**VAS**						
LBP	2.6 ± 2.5	1.3 ± 2.1	0.13	2.2 ± 2.0	1.9 ± 2.3	0.75
Lower leg pain	2.4 ± 2.9	1.7 ± 2.1	0.39	1.9 ± 1.7	2.3 ± 2.7	0.65
Lower leg numbness	2.5 ± 3.0	2.1 ± 2.0	0.62	1.9 ± 2.0	1.8 ± 2.6	0.91
LBP in motion	2.3 ± 2.4	1.4 ± 1.7	0.22	1.1 ± 1.3	1.7 ± 2.2	0.40
LBP in standing	2.7 ± 2.6	2.1 ± 1.6	0.36	2.9 ± 2.3	1.7 ± 2.2	0.28
LBP in sitting	2.5 ± 2.8	1.0 ± 1.4	0.029*	1.6 ± 1.8	1.1 ± 1.5	0.56
**JOA score**						
LBP	2.1 ± 0.8	2.6 ± 0.5	0.056	2.3 ± 0.5	2.6 ± 0.5	0.76
Lower leg pain	2.2 ± 0.8	2.6 ± 0.6	0.13	2.4 ± 0.7	2.6 ± 0.7	0.093
Intermittent claudication	2.3 ± 0.7	3.0 ± 0.0	0.0038*	2.4 ± 0.7	2.7 ± 0.5	0.42
ODI	27.7 ± 23.0	15.2 ± 11.4	0.030*	15.9 ± 8.5	17.8 ± 15.1	0.69
Nakai’s score	2.0 ± 0.9	2.8 ± 0.4	0.0089*	2.4 ± 0.7	2.7 ± 0.5	0.47
**Improvement**						
**VAS**						
LBP	3.4 ± 3.7	4.3 ± 3.2	0.48	4.6 ± 2.6	3.6 ± 2.8	0.43
Lower leg pain	4.4 ± 3.4	5.4 ± 3.4	0.42	5.2 ± 2.5	4.9 ± 3.5	0.78
Lower leg numbness	4.5 ± 3.8	3.5 ± 3.4	0.44	4.8 ± 3.0	4.5 ± 4.1	0.87
LBP in motion	3.2 ± 3.6	4.1 ± 3.2	0.44	4.5 ± 3.5	3.8 ± 3.5	0.67
LBP in standing	4.2 ± 4.4	4.5 ± 3.2	0.80	4.9 ± 2.8	5.3 ± 3.1	0.81
LBP in sitting	1.8 ± 4.6	3.6 ± 2.4	0.14	3.5 ± 3.0	3.7 ± 2.9	0.91
**JOA score**						
LBP	0.9 ± 0.9	1.5 ± 0.9	0.043*	1.3 ± 0.5	1.6 ± 0.8	0.25
Lower leg pain	1.5 ± 1.1	2.2 ± 0.9	0.063	1.6 ± 0.5	2.1 ± 1.0	0.27
Intermittent claudication	1.8 ± 1.1	2.5 ± 0.8	0.066	1.7 ± 1.0	1.8 ± 0.8	0.42
ODI	21.3 ± 28.4	28.9 ± 22.2	0.39	23.0 ± 16.1	23.2 ± 17.9	0.98

Data are presented as mean ± standard deviation.

Asterisks indicate statistically significant differences.

*R-ASD* radiological adjacent segment disease, *S-ASD* symptomatic adjacent segment disease, *Bony fusion rate (1Y, 5Y)* bony fusion rate at 1 year (or 5 years) postoperatively, *PI* pelvic incidence, *LL* lumbar lordosis, *DiLL* difference in preoperative LL (supine LL–standing LL), *VAS* visual analog scale, *JOA score* Japanese Orthopaedic Association score, *ODI* Oswestry disability index.

In PI-LL matched patients, no significant differences in the incidences of R-ASD, S-ASD, and bony fusion rates were found between the DiLL (+) and DiLL (−) groups. In addition, no significant differences in VASs, JOA, ODI, and Nakai’s scores were found between the DiLL (+) and DiLL (−) groups (Table 6).

## Discussion

Mid-term clinical data of patients treated with short-segment TLIF were analyzed, and preoperative DiLL was found to be useful for predicting mid-term clinical outcomes. Our results suggest that patients with DiLL (+) tend to show worse postoperative outcomes,with regard to LBP, lower-extremity pain, and gait disturbance. This tendency was more proounced in patients with PI-LL mismatch. In addition, there is a possibility that patients with DiLL (+) more frequently experienced S-ASD after short-segment TLIF, although the difference was not statistically significant.

Previously, a difference in LL depending on posture was reported^22,23^. Chevillotte et al. examined the lumbopelvic parameters of asymptomatic volunteers in standing and supine positions and reported that the mean LL values were greater in the standing position (54.8°) than in the supine position (50.2°)^23^. Park et al. examined patients with lumbar degenerative disease and found that the majority of them showed greater LL values in the standing position than in the supine position^22^. From these observations, the LL value in the standing position is typically slightly greater than that in the supine position, indicating that a normal DiLL is negative. Therefore, a positive DiLL value is considered abnormal. In the present study, we found that patients with DiLL (+) showed significantly smaller supine LL and standing LL and greater PI-LL and scoliotic curvature than patients with DiLL (−). However, the PI and lumbar flexibility were not significantly different between the two groups.

Patients with a positive DiLL may have functional disorders in maintaining the lumbar lordotic angle in the standing position and may not be able to maintain a normal standing position because of the dysfunction of supportive spinal tissues^24^ (such as intervertebral discs, facet joints, and muscles) or avoidance of pain-inducing postures^25,26^. Generally, neural element compression is relieved by lumbar flexion in patients with spinal stenosis. Thus, patients may develop a temporary sagittal plane deformity as compensation for neurogenic claudication^27,28^ These observations suggest that patients with positive DiLL have substantial dysfunction of the lumbar spine or severe neuropathic pain due to lumbar spinal stenosis. In contrast, this study revealed that the number of patients with scoliosis, a structural disorder of the lumbar spine, was significantly higher in the DiLL (+) group than in DiLL (−) group, suggesting that some patients in the DiLL (+) group had structural disorders. Functional and/or structural disorders may explain why the clinical outcomes after short-segment fusion surgery are expected to be unfavorable.

Previous studies have reported that a higher PI-LL value is associated with worse postoperative clinical outcomes and higher incidence of ASD after lumbar fusion surgery^7,9,10^. Ohyama et al. reported that DiLL is associated with short-term surgical outcomes after TLIF^13^. Moreover, the results of this study revealed that the mid-term clinical outcomes were significantly correlated with DiLL. In addition, after excluding patients with scoliosis, this study confirmed that DiLL is associated with postoperative clinical outcomes^29^. The subgroup analysis in this study revealed that the difference between patients with DiLL (+) and those with DiLL (−) was more evident in patients with PI-LL mismatch (PI-LL > 10°). Our results suggest that a combination of DiLL (+) and PI-LL > 10° is a strong predictor of worse mid-term outcomes, bucause both DiLL and PI-LL are related to lumbopelvic alignment. However, the two parameters have different implications. PI-LL is a static factor whereas DiLL is a dynamic factor that may reflect patients’ lumbar spinal function. In patients treated with long-segment spinal fusion surgery of the thoracic and lumbar spine, the lumbar spine is completely fused after surgery and dynamic factors may not be related to postoperative outcomes. In these patients, surgical outcomes may only be related to static factors including PI-LL^30,31^. We believe that the dynamic factor DiLL has a significant influence on surgical outcomes after short-segment fusion as some segments are not fused. It is reasonable to assume that the functional status of the non-fused segments influences surgical outcomes. Therefore, surgical results after short-segment TLIF are associated with both DiLL and PI-LL.

Currently, we cannot conclude that DiLL (+) mismatched patients should be treated with long-segment fusion surgery. However, spine surgeons should be aware that unfavorable surgical outcomes are expected after short-segment fusion in DiLL (+) mismatched patients and should carefully evaluate whether long-segment fusion surgery is more appropriate in such patients. Surgical outcome after short-segment lumbar fusion are associated with postoperative restoration of segmental lordosis^7,32,33^; therefore, spine surgeons should carefully choose the shape of fusion cages to increase segmental lordosis at the operated level in the treatment of DiLL (+) mismatched patients with short segment fusion surgery. Additionally, this will be helpful when DiLL (+) mismatched patients require revision surgery, such as kyphosis correction, after short-segment fusion.

Patients with a PI-LL > 10° were considered to have lumbar sagittal spinal malalignment^25,26^. Our study results suggest that the pathology of DiLL (+) mismatched patients is different from that of DiLL (−) mismatched patient. Patients with DiLL (+) mismatch may have severe functional or neurological disorders worsened by lumbar extension, both of which may prevent them from maintaining LL in the standing position. Lumbar extension may be limited in patients with DiLL (−) mismatch due to structural factors such as facet joint contractures, spinous process impingements, or hyperostosis, but not due to functional disorders. The difference in the surgical results may be due to the difference in the pathologies of DiLL (+)and DiLL (−) mismatches. We believe that DiLL (+) mismatch is mainly due to functional factors and partly due to structural factors in some patients, whereas DiLL (−) mismatch is mainly due to structural factors. In this study, both R-ASD and S-ASD increased in DiLL (+) patients, although the difference was not statistically significant. The increase in ASD in the DiLL (+) group may be partly explained by the finding that the number of patients with scoliosis was higher in the DiLL (+) group. However, the DiLL (+) group showed a non-significant tendency towards a higher incidence of S-ASD (p = 0.091), even when the analysis was limited to patients without scoliosis. At this moment, the possibility that functional disorders of the lumbar spine may increase the incidence of ASD in patients with DiLL (+) cannot be ruled out. Regarding bony fusion, favorable fusion rate can be expected after short segment-TLIF, regardless of preoperative DiLL value, as well as PI-LL value.

This study has several limitations. First, the sagittal vertical axis, an important factor influencing patient status, was not evaluated. However, whole-spine radiographs are not always obtained when patients are treated with short-segment TLIF. We believe that a simple factor that require no additional radiological examination is ideal for clinical use. Even when CT is not performed preoperatively, DiLL can be evaluated using supine radiography or magnetic resonance imaging. Therefore, DiLL is ideal for predicting postoperative outcomes. Second, there were significant differences in patient age, preoperative PI-LL, and scoliotic curvature between groups. Multiple regression analysis was performed to exclude the influence of age, sex, BMI, scoliosis, and number of fused segments and a significant correlation was found between DiLL and each clinical score. As DiLL and PI-LL were found to be significantly correlated^34^, the two values could not be used in the multiple regression analysis to avoid multicollinearity. However, this study revealed that the difference in outcomes between patients with DiLL (+) and those with DiLL (−) was more evident in patients with PI-LL mismatches. These results indicate that the combination of DiLL and PI-LL is a strong predictor of the postoperative course after short-segment TLIF. Regarding scoliotic curvature, a comparison analysis between the DiLL (+) and DiLL (−) groups was performed after excluding patients with scoliotic curavature > 10°. It revealed significant differences in postoperative outcomes between the two groups. This result indicates that DiLL is an independent factor predictor of postoperative outcomes irrespective of scoliosis. Third, the number of patients included in the study was limited. However, this study successfully demonstrated a significant difference in clinical outcomes between the DiLL (+) and DiLL (−) groups. The incidence of postoperative S-ASD was higher in the DiLL (+) group; however, the difference was not statistically significant. Moreover, when performing comparative analysis by dividing patients into four groups by DiLL and PI-LL, the number of patients in each group was limited, suggesting that the analysis was underpowered. Future studies with more patients are warranted to more precisely investigate the incidence of S-ASD between the groups and the influence of a combination of DiLL and PI-LL on the postoperative outcomes. Fourth, there are no background data supporting the appropriateness of classifying patients into DiLL (+) and DiLL (−) groups because DiLL is a newly proposed lumbopelvic parameter^13^. In the future, further studies are needed to clarify the cut-off value for DiLL for classifying patients with normal or abnormal lumbar function.

**Figure 2:**
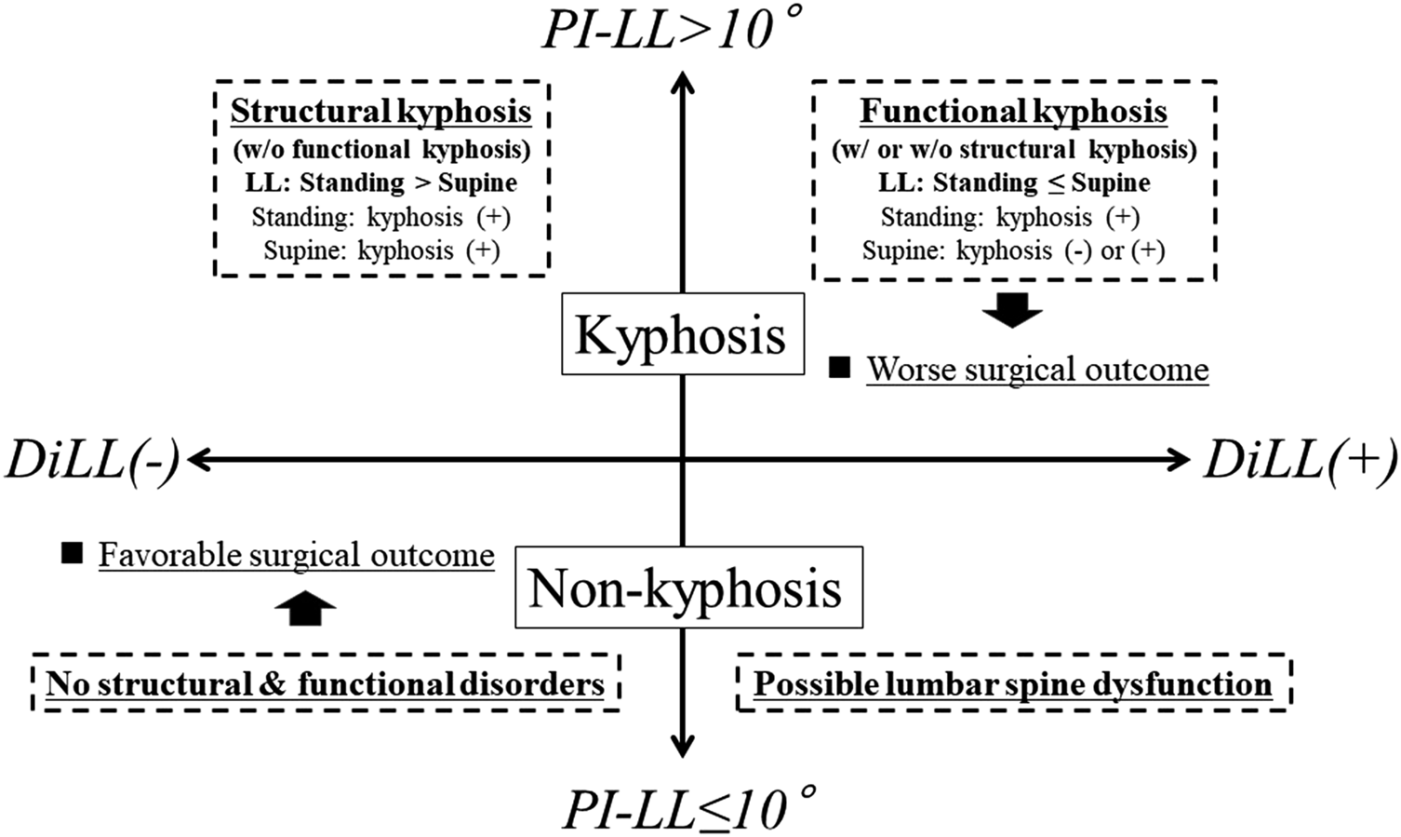
The predicted pathologies of patients in the four difference in lumbar lordosis (DiLL) and pelvic incidence minus LL (PI-LL) subgroups are shown. Mismatch was defined as PI-LL > 10°, and lumbar spine dysfunction was defined as DiLL (+). In patients with PI-LL > 10°, mismatch is mainly due to functional factors when DiLL is positive and structural factors when DiLL is negative. Worse surgical outcomes and subsequent surgeries were expected in patients with PI-LL > 10° and DiLL (+).

In conclusion, higher DiLL values were correlated with poorer patient outcomes after TLIF. Poor outcomes were expected in DiLL (+) patients, particularly in those with preoperative PI-LL mismatch (PI-LL > 10°) (Fig. 2). DiLL is a simple parameter that can be easily measured using standing radiography and supine CT. Instead of CT, MRI or lateral radiographs obtained in the supine position can be used to measure the DiLL value. Thus, preoperative evaluation of DiLL (dynamic factor) and PI-LL (static factor) is recommended because it is useful for predicting mid-term postoperative outcomes in patients who undergo short-segment TLIF (Fig. 2).

## Data Availability

The datasets generated and/or analyzed during the current study are available from the corresponding author on reasonable request.
